# The maintenance of cisplatin- and paclitaxel-induced mechanical and cold allodynia is suppressed by cannabinoid CB_2_ receptor activation and independent of CXCR4 signaling in models of chemotherapy-induced peripheral neuropathy

**DOI:** 10.1186/1744-8069-8-71

**Published:** 2012-09-22

**Authors:** Liting Deng, Josée Guindon, V Kiran Vemuri, Ganesh A Thakur, Fletcher A White, Alexandros Makriyannis, Andrea G Hohmann

**Affiliations:** 1 Program in Neuroscience, Indiana University, Bloomington, IN, USA; 2 Department of Molecular and Cellular Biochemistry, Indiana University, Bloomington, IN, USA; 3 Interdisciplinary Biochemistry Graduate Program, Indiana University, Bloomington, IN, USA; 4 Department of Psychological and Brain Sciences, Indiana University, Bloomington, IN, USA; 5 Center for Drug Discovery, Bouvé College of Health Sciences, Northeastern University, Boston, MA, USA; 6 Department of Anesthesia, Indiana University School of Medicine, Indianapolis, IN, USA

**Keywords:** Endocannabinoid, Cannabilactone, AM1710, Chemotherapy, Neuropathic pain, Chemokine, CXCR4, Mechanical allodynia, Cold allodynia, Hyperalgesia

## Abstract

**Background:**

Chemotherapeutic agents produce dose-limiting peripheral neuropathy through mechanisms that remain poorly understood. We previously showed that AM1710, a cannabilactone CB_2_ agonist, produces antinociception without producing central nervous system (CNS)-associated side effects. The present study was conducted to examine the antinociceptive effect of AM1710 in rodent models of neuropathic pain evoked by diverse chemotherapeutic agents (cisplatin and paclitaxel). A secondary objective was to investigate the potential contribution of alpha-chemokine receptor (CXCR4) signaling to both chemotherapy-induced neuropathy and CB_2_ agonist efficacy.

**Results:**

AM1710 (0.1, 1 or 5 mg/kg i.p.) suppressed the maintenance of mechanical and cold allodynia in the cisplatin and paclitaxel models. Anti-allodynic effects of AM1710 were blocked by the CB_2_ antagonist AM630 (3 mg/kg i.p.), but not the CB_1_ antagonist AM251 (3 mg/kg i.p.), consistent with a CB_2_-mediated effect. By contrast, blockade of CXCR4 signaling with its receptor antagonist AMD3100 (10 mg/kg i.p.) failed to attenuate mechanical or cold hypersensitivity induced by either cisplatin or paclitaxel. Moreover, blockade of CXCR4 signaling failed to alter the anti-allodynic effects of AM1710 in the paclitaxel model, further suggesting distinct mechanisms of action.

**Conclusions:**

Our results indicate that activation of cannabinoid CB_2_ receptors by AM1710 suppresses both mechanical and cold allodynia in two distinct models of chemotherapy-induced neuropathic pain. By contrast, CXCR4 signaling does not contribute to the maintenance of chemotherapy-induced established neuropathy or efficacy of AM1710. Our studies suggest that CB_2_ receptors represent a promising therapeutic target for the treatment of toxic neuropathies produced by cisplatin and paclitaxel chemotherapeutic agents.

## Background

More than half of cancer patients are treated with chemotherapeutic agents (e.g. platinum-based compounds (cisplatin), taxanes (paclitaxel) and vinca alkaloids (vincristine)), of which about 30-40% patients develop neuropathic pain [1-4]. Chemotherapy-induced neuropathy is dose-limiting and is the major toxicity responsible for discontinuation of chemotherapy [3,5-7]. Severe peripheral neuropathy can occur at the early stage of chemotherapy and persist for years after cessation of treatment [8]. Sensory abnormalities (such as tingling, numbness) as well as shooting and burning pain due to chemotherapy can impair the quality of life in patients [2]. To date, no medication has been recognized to effectively and safely cure chemotherapy-induced neuropathy [6,9,10].

Cannabinoids suppress pain through activation of cannabinoid CB_1_ and/or CB_2_ receptors [11]. Cannabis-based medicines, such as Cesamet® (nabilone) or Sativex® (mixture of Δ^9^-tetrahydrocannabinol and non-psychoactive cannabidiol), are already used clinically to manage neuropathic pain [12,13]. However, cannabinoids may produce unwanted central nervous system (CNS) side effects associated with CB_1_ receptors. Efficacy of cannabis medicines in treating chemotherapy-induced neuropathy has yet to be fully evaluated [13]. A small number of preclinical studies have reported a role of CB_2_-selective agonists in suppressing chemotherapy-evoked neuropathic pain [14-17]. In these studies, CB_2_-selective agonists suppressed paclitaxel- or vincristine-induced mechanical allodynia [14-18]. Whether CB_2_ selective agonists suppress cold allodynia after development of chemotherapy-induced neuropathic pain remains poorly understood and effects of CB_2_-selective agonists on cisplatin-induced neuropathy are unknown.

**Figure 1:**
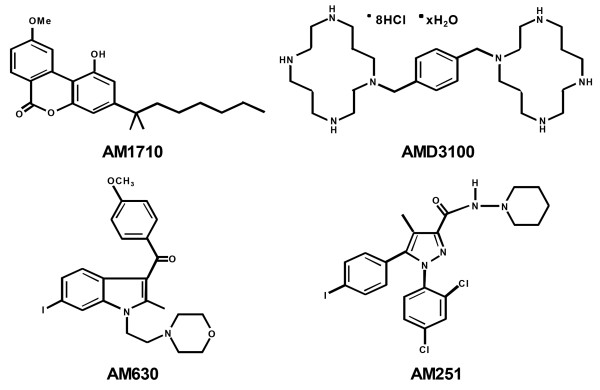
**Chemical stuctures of compounds employed.** Chemical structure of the cannabilactone CB_2_ agonist AM1710, the CXCR4 antagonist AMD3100, the CB_2_ receptor antagonist AM630 and the CB_1_ receptor antagonist AM251.

AM1710, a cannabilactone CB_2_ agonist with limited blood brain barrier penetration [19], exhibits 54-fold selectivity for CB_2_ over CB_1_ receptors [20]. We previously showed that AM1710 produces antinociception in the plantar test in naïve animals without producing CNS side effects, such as hypothermia, hypoactivity, tail-flick antinociception or motor ataxia at doses 100 times higher than the lowest maximally effective antinociceptive dose [19]. In the present study, we evaluated effects of AM1710 (Figure 1) in two distinct animal models of chemotherapy-induced neuropathy (cisplatin and paclitaxel models) and characterized its mechanism of action. Pharmacological specificity was established using cannabinoid CB_2_ (AM630) and CB_1_ (AM251) antagonists (Figure 1).

Mechanisms underlying chemotherapy-induced neuropathy remain poorly understood [21]. An emerging body of literature implicates a role for chemokine stromal derived factor-1 (SDF-1/CXCL12) and its receptor CXCR4 in mechanisms of other distinct neuropathic pain states [22]. For instance, blockade of CXCR4 signaling by its antagonist AMD3100 reversed the maintenance of neuropathic pain induced by either chronic constriction injury (CCI) of the sciatic nerve [23] or HIV-associated neuropathy [24,25]. However, whether CXCR4 signaling is also involved in chemotherapy-induced neuropathy has not been studied. In the present study, we investigated the role of CXCR4 signaling in established chemotherapy-induced neuropathic pain and examined its potential interaction with CB_2_ signaling.

## Results

### Established chemotherapy-induced neuropathy

Prior to cisplatin or paclitaxel treatment, there were no differences between groups in either paw withdrawal thresholds to mechanical stimulation or paw withdrawal frequencies to cold stimulation (*P* > 0.15 for all studies).

**Figure 2:**
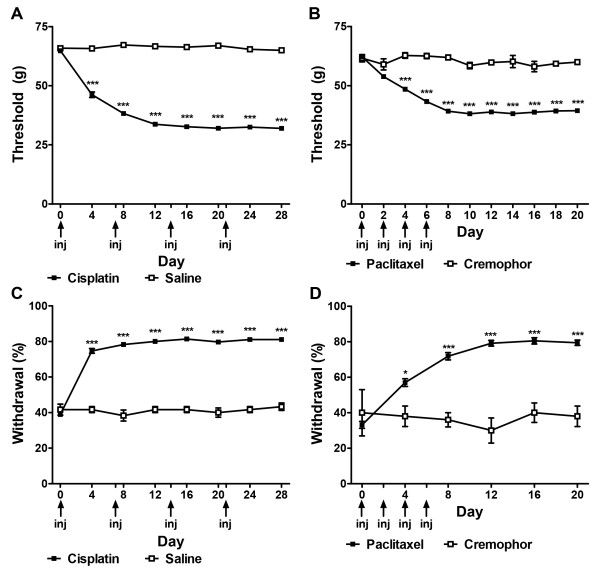
**Time course of development of chemotherapy-induced peripheral neuropathy evoked by cisplatin and paclitaxel treatment.** Mechanical (**A**, **B**) and cold (**C**, **D**) allodynia developed following cisplatin (**A**, **C**) or paclitaxel (**B**, **D**) treatment. Arrows show timing of injections of chemotherapeutic agents. Data are expressed as mean ± SEM (paclitaxel, n = 76; cremophor, n = 5; cisplatin, n = 36; saline, n = 6). ^***^P < 0.001, ^**^P < 0.01, ^*^P < 0.05 vs. vehicle. Non-chemotherapy controls received saline in lieu of cisplatin (**A**, **C**) or cremophor vehicle in lieu of paclitaxel (**B**, **D**), repeated measures ANOVA and One-way ANOVA at each time point.

Cisplatin or paclitaxel treatment established and maintained neuropathic states characterized by hypersensitivities to mechanical and cold stimulation. Cisplatin decreased mechanical paw withdrawal thresholds (F_1,40_ = 1565.23, *P* < 0.0001; Figure 2A) and increased cold withdrawal frequencies (F_1,40_ = 632.24, *P* < 0.0001; Figure 2C). Mechanical (*P* < 0.0001) and cold (*P* < 0.0001) allodynia were maintained from day 4 to day 28 in cisplatin-treated group (Figure 2A and C). Similarly, paclitaxel decreased paw withdrawal thresholds to mechanical stimulation (F_1,79_ = 290.19, *P* < 0.0001; Figure 2B) and increased frequencies of withdrawal to cold stimulation (F_1,79_ = 37.11, *P* < 0.0001; Figure 2D). Mechanical (*P* < 0.0001) and cold (*P* < 0.03) allodynia were present from day 4 to day 20 in the paclitaxel-treated group (Figure 2B and D).

### AM1710 suppressed the maintenance of mechanical and cold allodynia produced by either cisplatin or paclitaxel treatment

**Figure 3:**
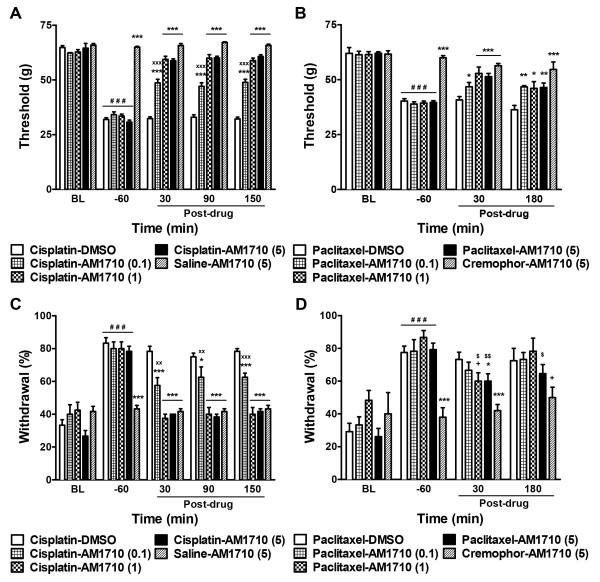
**Effect of AM1710 on chemotherapy-induced mechanical and cold allodynia.** AM1710 suppressed both mechanical (**A**, **B**) and cold (**C**, **D**) allodynia evoked by cisplatin (**A**, **C**) or paclitaxel (**B**, **D**) treatment. Data are expressed as mean ± SEM (n = 5-13 per group). ^***^P < 0.001, ^**^P < 0.01, ^*^P < 0.05 vs. DMSO vehicle, One-way ANOVA followed by Dunnett *post hoc* test. ^xxx^*P* < 0.001 vs. AM1710 (1 and 5 mg/kg i.p.), One-way ANOVA followed by Bonferroni *post hoc* test. ^+^*P* < 0.05 vs. DMSO vehicle, planned comparison t-test. ^$$^*P* < 0.01, ^$^*P* < 0.05 vs. pre-drug baseline (−60 min), paired t-test. ^###^*P* < 0.001 vs. pre-cisplatin/paclitaxel baseline (BL), repeated measures ANOVA.

The cannabilactone AM1710 (0.1, 1 and 5 mg/kg i.p.) suppressed cisplatin-evoked mechanical (F_4,21_ = 547.02, *P* < 0.0001) and cold (F_4,21_ = 59.10, *P* < 0.0001) allodynia compared to vehicle treatment (Figure 3A and C). AM1710 (1 or 5 mg/kg i.p.) fully reversed cisplatin-evoked neuropathy and normalized responses to pre-drug levels for both modalities (*P* = 0.10 mechanical, Figure 3A; *P* = 0.17 cold, Figure 3C). The lowest dose of AM1710 (0.1 mg/kg i.p.) suppressed mechanical (*P* < 0.0001 vs. 1 or 5 mg/kg i.p.) and cold (*P* < 0.002 vs. 1 or 5 mg/kg i.p.) allodynia to a lesser extent than either the middle or the high doses at each time point.

AM1710 produced time-dependent attenuations of cisplatin-evoked mechanical (F_16,84_ = 62.38, *P* < 0.0001) and cold (F_16,84_ = 15.52, *P* < 0.0001) allodynia (Figure 3A and C). Anti-allodynic effects of AM1710 on cisplatin-evoked mechanical (*P* < 0.0001) and cold (*P* < 0.02) responsiveness lasted at least 150 min post injection. AM1710 failed to alter responsiveness to mechanical (*P* = 0.13) or cold (*P* = 0.94) stimulation in animals treated with saline vehicle in lieu of cisplatin (Figure 3A and C).

AM1710 (1 and 5 mg/kg i.p.) also suppressed paclitaxel-evoked mechanical (F_4,37_ = 13.75, *P* < 0.0001) and cold (F_4,37_ = 4.41, *P* < 0.01) allodynia compared to the vehicle group (Figure 3B and D). The low dose of AM1710 (0.1 mg/kg i.p.) suppressed paclitaxel-evoked mechanical (*P* < 0.05) allodynia but did not reliably attenuate cold allodynia (*P* = 0.44). AM1710 produced time-dependent suppressions of paclitaxel-induced mechanical (F_12,111_ = 7.09, *P* < 0.0001) and cold (F_12,111_ = 3.15, *P* < 0.001) hypersensitivities. Suppression of mechanical allodynia was observed relative to vehicle (Figure 3B) throughout the 180 min post injection observation interval (*P* < 0.05). AM1710 (1–5 mg/kg i.p.) attenuated paclitaxel-evoked cold allodynia relative to vehicle at 30 min post injection (*P* < 0.04; Figure 3D). AM1710 (5 mg/kg i.p.) also reliably attenuated paclitaxel-evoked cold allodynia relative to pre-drug baseline levels at both 30 (*P* < 0.03) and 180 (*P* < 0.04) min post injection (Figure 3D). AM1710 failed to alter responsiveness to mechanical (*P* = 0.06) or cold (*P* = 0.72) stimulation in animals treated with cremophor vehicle in lieu of paclitaxel (Figure 3B and D).

**Figure 4:**
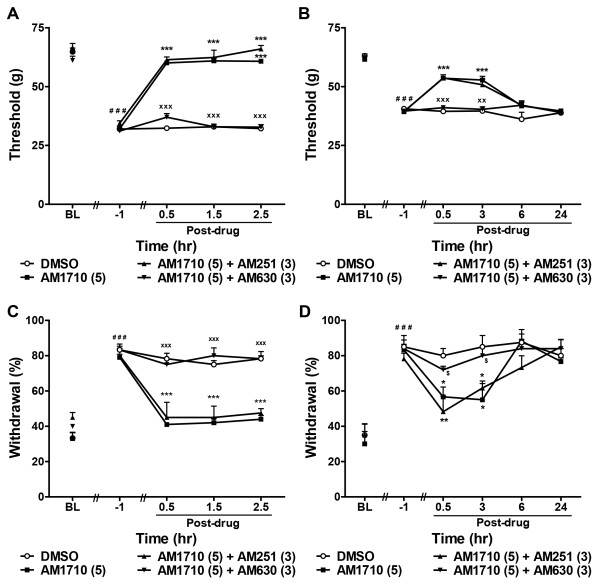
**The cannabilactone AM1710 suppresses chemotherapy-induced mechanical and cold allodynia through a CB**_**2**_**-specific mechanism.** AM1710-induced suppressions of cisplatin- (**A**, **C**) and paclitaxel- (**B**, **D**) evoked mechanical (**A**, **B**) and cold (**C**, **D**) allodynia were blocked by the CB_2_ antagonist AM630 (3 mg/kg i.p.) but not the CB_1_ antagonist AM251 (3 mg/kg i.p.). Data are expressed as mean ± SEM (n = 5-6 per group). ^***^P < 0.001, ^**^P < 0.01, ^*^P < 0.05 vs. DMSO vehicle, One-way ANOVA followed by Dunnett *post hoc* test. ^xxx^*P* < 0.01, ^xx^*P* < 0.01, ^x^P < 0.05 vs. AM1710 (5 mg/kg i.p.), Bonferroni *post hoc* test. ^$^*P* < 0.05 vs. AM1710 (5 mg/kg i.p.), planned t-test. ^###^*P* < 0.001 vs. pre-cisplatin/paclitaxel baseline (BL), repeated measures ANOVA.

To examine the duration of action of AM1710 (5 mg/kg i.p.), a subset of paclitaxel-treated animals was tested at 6 and 24 h post injection. AM1710 reliably suppressed mechanical (*P* < 0.0001) and cold (*P* < 0.03) allodynia over 180 min post injection (Figure 4B and D). Mechanical (*P* = 0.43) and cold (*P* = 0.76) allodynia was reinstated 6 h post injection (Figure 4B and D). Similarly, no evidence for anti-allodynic efficacy of AM1710 (0.1-5 mg/kg i.p.) was found at 6 h following injection in the subset of cisplatin-treated animals used to further characterize the time course of AM1710 antinociception (data not shown).

### Anti-allodynic effects of AM1710 were mediated by cannabinoid CB_2_ receptors

To evaluate pharmacological specificity, the highest behaviorally active dose of AM1710 (5 mg/kg i.p.) was co-administered with either the CB_2_ antagonist AM630 (3 mg/kg i.p.) or the CB_1_ antagonist AM251 (3 mg/kg i.p.) in cisplatin- or paclitaxel-treated animals.

Anti-allodynic effects of AM1710 on cisplatin-evoked mechanical (F_3,22_ = 311.81, *P* < 0.0001) and cold (F_3,22_ = 39.03, *P* < 0.0001) hypersensitivities were blocked by the CB_2_ antagonist AM630 throughout the 150 min post injection observation interval (*P* < 0.0001 mechanical and *P* < 0.0001 cold; Figure 4A and C). By contrast, the CB_1_ antagonist AM251 failed to block the mechanical (*P* = 1.00) and cold (*P* = 1.00) anti-allodynic effects of AM1710 (Figure 4A and C).

Similarly, in the paclitaxel model, the CB_2_ antagonist AM630 blocked the AM1710-induced suppressions of mechanical (F_3,17_ =12.73, *P* < 0.0001) and cold (F_3,17_ = 3.20, *P* < 0.05) allodynia from 30 to 180 min post injection (*P* < 0.001 mechanical, Figure 4B; and *P* < 0.03 cold, Figure 4D). Hypersensitivities to mechanical (*P* = 0.87) and cold (*P* = 0.41) stimulation were reinstated by 6 h post injection (Figure 4B and D). By contrast, the CB_1_ antagonist AM251 failed to block the AM1710-induced suppressions of mechanical (*P* = 1.00) and cold (*P* = 1.00) allodynia (Figure 4B and D).

**Figure 5:**
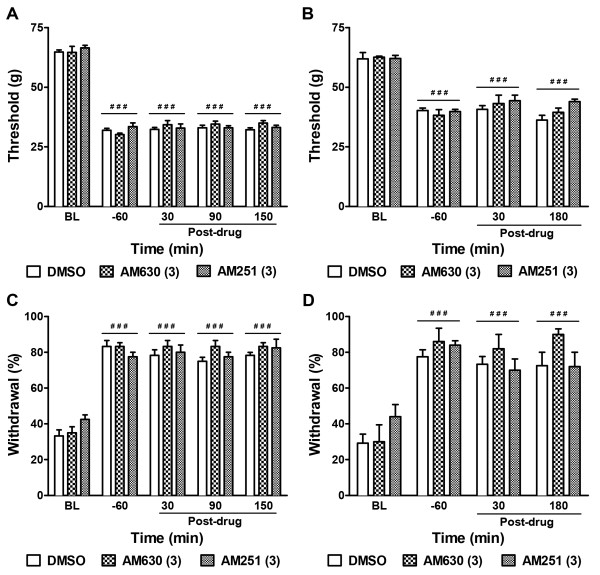
**Pharmacological blockade of CB**_**2 **_**or CB**_**1 **_**receptors does not alter cisplatin- or paclitaxel-induced neuropathic nociception.** Neither the CB_2_ antagonist AM630 (3 mg/kg i.p.) nor the CB_1_ antagonist AM251 (3 mg/kg i.p.) altered cisplatin- (**A**, **C**) or paclitaxel- (**B**, **D**) evoked mechanical (**A**, **B**) and cold (**C**, **D**) allodynia. ^###^*P* < 0.001 vs. baseline (BL) prior to chemotherapy treatment, repeated measures ANOVA.

Antagonist treatment alone failed to alter nociceptive thresholds in cisplatin- or paclitaxel-treated animals. Neither the CB_2_ antagonist AM630 (3 mg/kg i.p.) nor the CB_1_ antagonist AM251 (3 mg/kg i.p.), administered alone, altered mechanical paw withdrawal thresholds (F_2,13_ = 0.38, *P* = 0.69) or cold withdrawal frequencies (F_2,13_ = 3.32, *P* = 0.07) relative to vehicle in cisplatin-treated animals at any time point (F_8,52_ =1.35, *P* = 0.24 mechanical, Figure 5A; F_8,52_ =1.10, *P* = 0.38 cold, Figure 5C). Similarly, the same doses of AM630 and AM251, administered alone, failed to alter paclitaxel-evoked mechanical (F_2,19_ = 0.89, *P* = 0.43) and cold (F_2,19_ = 0.88, *P* = 0.43) allodynia relative to vehicle at any time point (F_6,57_ =1.19, *P* = 0.33 mechanical, Figure 5B; F_6,57_ =1.44, *P* = 0.22 cold, Figure 5D).

### Blockade of CXCR4 signaling with AMD3100 failed to reverse established chemotherapy-evoked neuropathy

**Figure 6:**
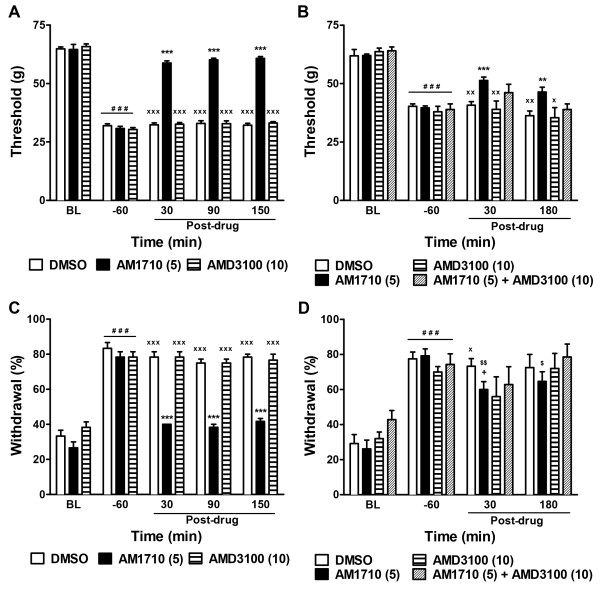
**CXCR4 signaling did not contribute to either chemotherapy-induced neuropathy or CB**_**2 **_**agonist efficacy.** The CXCR4 antagonist AMD3100 (10 mg/kg i.p.) failed to suppress the maintenance of cisplatin- (**A**, **C**) or paclitaxel- (**B**, **D**) evoked mechanical (**A**, **B**) and cold (**C**, **D**) allodynia. The CXCR4 antagonist AMD3100 (10 mg/kg i.p.) did not alter antinociceptive efficacy of the CB_2_ agonist AM1710 (5 mg/kg i.p.) in suppressing paclitaxel-induced mechanical (**B**) and cold (**D**) allodynia. ^***^P < 0.001, ^**^P < 0.01, ^*^P < 0.05 vs. DMSO vehicle, One-way ANOVA followed by Dunnett *post hoc* test. ^+^*P* < 0.05 vs. DMSO vehicle, planned t-test. ^xxx^P < 0.001, ^xx^*P* < 0.01, ^x^*P* < 0.05 vs. AM1710 (5 mg/kg), Bonferroni *post hoc* test. ^$$^*P* < 0.01, ^$^*P* < 0.05 vs. pre-drug baseline (−60 min), paired t-test. ^###^*P* < 0.001 vs. baseline (BL) prior to cisplatin/paclitaxel treatment, repeated measures ANOVA.

We asked whether blockade of CXCR4 signaling with its antagonist AMD3100 would reverse established neuropathy induced by cisplatin and paclitaxel treatment. Whereas AM1710 (5 mg/kg i.p.) attenuated mechanical and cold allodynia in both neuropathy models, AMD3100 (10 mg/kg i.p.) failed to do so. In the cisplatin model, AMD3100 (10 mg/kg i.p.) failed to alter mechanical (*P* = 0.97, *P* = 0.99 and *P* = 0.59 at 30, 90 and 150 min, respectively) or cold (*P* = 1.00, *P* = 1.00 and *P* = 0.84 at 30, 90 and 150 min, respectively) allodynia relative to vehicle (Figure 6A and C). Similarly, in paclitaxel-treated animals, the same dose of AMD3100 failed to alter mechanical withdrawal thresholds (*P* = 0.93 and *P* = 0.99 at 30 and 180 min, respectively) or cold withdrawal frequencies (*P* = 0.13 and *P* = 1.00 at 30 and 180 min, respectively) compared to vehicle (Figure 6B and D).

### Blockade of CXCR4 signaling with AMD3100 failed to enhance CB_2_ agonist efficacy

We asked whether blockade of CXCR4 signaling in the paclitaxel model would enhance the efficacy of a CB_2_ agonist by assessing the effects of co-administration of AMD3100 (10 mg/kg i.p.) with a sub-maximal dose of AM1710 (5 mg/kg i.p.). Responsiveness to mechanical (*P* = 0.61 and *P* = 0.23 at 30 and 180 min, respectively) and cold (*P* = 1.00 and *P* = 0.86 at 30 and 180 min, respectively) stimulations did not differ in animals receiving AM1710 in either the presence or absence of AMD3100 (Figure 6B and D).

## Discussion

To our knowledge, this is the first study to demonstrate antinociceptive efficacy of a CB_2_-selective agonist in the cisplatin model and the first to evaluate a potential role for CXCR4 signaling in contributing to mechanisms of chemotherapy-induced peripheral neuropathy. Our studies demonstrate that activation of cannabinoid CB_2_ receptors suppresses both mechanical and cold allodynia induced by either cisplatin or paclitaxel treatment. Effects of the cannabilactone CB_2_-selective agonist AM1710 on established neuropathy induced by chemotherapeutic treatment have not previously been evaluated. We recently showed that systemic administration of AM1710 in naïve animals produces antinociception to heat, but not to mechanical stimulation, in the plantar test in otherwise naïve animals [19]. Suppression of thermal nociception was also observed following local (i.paw) administration of AM1710 [20]. In the present study, we extended our investigation of the antinociception profile of AM1710 by showing that AM1710 suppressed both mechanical and cold allodynia in two distinct models of chemotherapy-induced neuropathy. Strikingly, anti-allodynic effects of AM1710 were observed at low doses (0.1 mg/kg i.p.) and lasted at least 2.5-3 h following systemic administration. In our previous work, higher doses of AM1710 (10 mg/kg i.p.; a dose 100 times higher than the lowest effective antinociceptive dose identified here) failed to produce CNS side-effects in otherwise naïve animals [19]. These observations suggest that AM1710 exhibits a very favorable efficacy to toxicity ratio.

In the present study, AM1710-induced suppressions of chemotherapy-induced mechanical and cold allodynia were completely blocked by the CB_2_ antagonist AM630 (3 mg/kg i.p.), but not the CB_1_ antagonist AM251 (3 mg/kg i.p.), following either cisplatin or paclitaxel treatment. Thus, selective activation of cannabinoid CB_2_ receptors attenuates the maintenance of neuropathic pain induced by diverse chemotherapeutic agents. These findings are consistent with other studies showing that CB_2_ selective agonists are antinociceptive in animal models of toxic neuropathies [14-17,26]. In particular, AM1714, which also belongs to the cannabilactone class of CB_2_ selective agonists [20], suppresses paclitaxel-induced mechanical allodynia via a CB_2_-specific mechanism [14]. In this latter study, responses to cold stimulation were not previously characterized [14]. CB_2_ receptors are found primarily, although not exclusively, in cells of the immune system and reside centrally at low levels relative to CB_1_ receptors [27,28]; however, CB_2_ receptor expression is highly inducible in response to injury (for review see [29]). CB_2_ receptors have also been localized to primary afferent terminals in humans [30]. Thus, it may be possible to target the cannabinoid CB_2_ signaling system to produce antinociception without adverse CNS side effects associated with CB_1_ receptors (e.g. HU308 [31], AM1241 [32], and AM1710 [19,33]). Our studies suggest that cannabinoid CB_2_ receptors represent a promising target for the treatment of toxic neuropathies induced by chemotherapeutic agents.

Cisplatin, a platinum-based compound, produces sensory axonal nerve damage and paresthesias in the distal extremities in humans [34,35]. Paclitaxel belongs to the taxane class of chemotherapeutic agents and also produces peripheral nerve damage and sensory neuropathies such as numbness, tingling and burning pain in patients [36]. Different mechanisms may underly the maintenance of neuropathy induced by different classes of chemotherapeutic agents (for review see [21]); however, similarities are also shared between the models. For example, glutamatergic neurotransmission and N-methyl-D-aspartate (NMDA) receptors are involved in both cisplatin- and paclitaxel-induced neuropathic pain [21]. Peripheral nerve damage results in glutamate/NMDA receptor-mediated sensitization and spontaneous activity of primary afferents, and causes hyper-excitability of dorsal horn neurons [37]. Decreased glutamate levels facilitate nerve conduction in the cisplatin and paclitaxel models [38]. The NMDA receptor antagonist ketamine also produces antinociceptive effects in paclitaxel-treated rats [39]. Paclitaxel treatment also down-regulates glial glutamate transporters (i.e. GLAST and GLT-1) in the spinal dorsal horn [40]. The transient receptor potential channel family is also implicated in mechanisms of nociception in both models. Cisplatin increases transient receptor potential vanilloid 1 (TRPV1) and transient receptor potential ankyrin 1 (TRPA1) expression levels and nociceptor responsiveness in dorsal root ganglion (DRG) neurons [41]. Mechanical hypersensitivity is preserved in cisplatin-treated animals lacking TRPV1 [41] and reduced in paclitaxel-treated animals lacking TRPV4 [42]. In addition, according to the mitotoxicity hypothesis, both cisplatin and paclitaxel induce morphological changes (swollen and vacuolated mitochondria) and dysfunction (reduced respiration and energy production) of mitochondria in axons, which then alters intracellular calcium levels and initiates apoptosis pathways [43-46]. CB_2_ agonists may interfere with pro-nociceptive signaling cascades (i.e. NMDA, TRPV1, TRPA1) activated by the chemotherapy. More work is necessary to determine whether CB_2_ agonists such as AM1710 directly reduce nociceptor excitability (i.e. at the level of the primary afferent terminal or DRG) and/or attenuate the mitotoxicity and structural damage to DRG or axons that are produced by chemotherapy and result in aberrant neuronal excitability [47-50].

In the present study, AM1710 suppressed the hypersensitivities induced by both cisplatin and paclitaxel through a CB_2_-specific mechanism, suggesting a shared role for CB_2_ in modulating hypersensitivity in both models. CB_2_ modulation of chemotherapy-induced neuropathy may result from suppression of central sensitization. In animal models of inflammatory pain and nerve injury, CB_2_ agonists (such as AM1241 and JWH133) decrease windup and central sensitization [48] as well as mechanically-evoked responses [49,50] in spinal dorsal horn neurons (for review see [29]). Peripheral nerve injury also leads to secretion of chemokines, increased release of proinflammatory cytokines, and increased activation of microglia and astrocytes, which facilitate responses to noxious stimulations and contribute to central sensitization (for review see [51]). Hence, it is possible that activation of CB_2_ receptors attenuates chemotherapy-induced neuropathy by interfering with astrocyte and/or microglial activation and pro-inflammatory cytokine signaling [16,40,52,53].

To better explore the maintenance of chemotherapy-induced neuropathic pain and its modulation by CB_2_ agonists, we investigated the possible contribution of the alpha-chemokine receptor CXCR4 to cisplatin and paclitaxel-induced neuropathies. CXCR4 signaling has been implicated in the mechanisms underlying several neuropathic pain states. Notably, intradermal injection of SDF-1α in rats produces onset tactile allodynia, suggesting a direct role in pain [54]. Blockade of CXCR4 signaling by its antagonist AMD3100 also suppresses established mechanical allodynia in HIV-associated peripheral neuropathy [24,25] and reverses heat hyperalgesia, but not mechanical allodynia, induced by chronic constriction injury of sciatic nerve (CCI) [23]. Contrary to HIV-associated neuropathy, blockade of CXCR4 signaling by AMD3100 in our study did not inhibit the maintenance of mechanical or cold allodynia evoked by either cisplatin or paclitaxel. Thus, CXCR4 signaling is unlikely to contribute to the maintenance of neuropathic pain induced by chemotherapeutic agents. Moreover, chemotherapy-induced neuropathy is thus likely to recruit pain mechanisms distinct from traumatic nerve injury (e.g. CCI) and HIV-associated neuropathy. Physiological studies indicate that neuropathic pains induced by traumatic nerve injury produce axonal degeneration with an increased discharge in A-fiber and C-fiber nociceptors [55,56]. By contrast, this degeneration is not observed in animals treated with paclitaxel or vincristine [43,57,58], although hypersensitivities of C-fiber nociceptors are nonetheless observed [59,60]. Indeed, paclitaxel increases spontaneous discharge in both A-fibers and C-fibers [61]. Diverse second messengers, including protein kinase C_ɛ_ and protein kinase A, also contribute to the maintenance of paclitaxel-induced hyperalgesia. For example, intradermal injection of antagonists for protein kinase A attenuates hyperalgesia evoked by both acute and chronic paclitaxel treatments [62]. More work is necessary to elucidate mechanisms of chemotherapy-induced neuropathic pain at the molecular and neurophysiological levels and characterize effects of CB_2_ agonists such as AM1710 on nociceptor excitability in these models.

Our study is the first to test the hypothesis that CB_2_ modulation of chemotherapy-induced neuropathy may interact with CXCR4 signaling. Several publications have suggested that CXCR4 signaling crosstalks with the cannabinoid system. Behavioral and physiological studies suggest that both antinociceptive and hypothermic effects of the mixed CB_1_/CB_2_ agonist WIN55,212-2 is inhibited by CXCR4 activation with SDF-1α [63,64]. The interaction between the CXCR4 and the cannabinoid CB_2_ receptor signaling also modulates chemotaxis of CD4+ T lymphocytes [65] as well as growth and metastasis of breast cancer [66]. Although there is support for spinal cord-infiltrating CD4+ T lymphocytes in contributing to neuropathic pain due to spinal nerve transection injury [67], chemotherapy-induced neuropathy does not appear to be influenced by either this cell type or the CXCR4 antagonist AMD3100. Furthermore, blockade of CXCR4 signaling did not reliably enhance (or inhibit) the anti-allodynic efficacy of AM1710. These results imply that CXCR4 signaling does not contribute to CB_2_-modulation of established chemotherapy-induced neuropathy. More work is necessary to determine whether CXCR4 signaling contributes to the development of chemotherapy-induced neuropathy.

## Conclusions

In conclusion, the present study demonstrates that selective activation of cannabinoid CB_2_ receptors suppresses neuropathic nociception to multiple stimulus modalities that is evoked by different classes of chemotherapeutic agents. The cannabilactone CB_2_ selective agonist AM1710 produces CB_2_-mediated suppressions of mechanical and cold allodynia induced by chemotherapeutic treatment with cisplatin or paclitaxel. In addition, our data indicate that neither the maintenance of chemotherapy-induced neuropathy nor the anti-allodynic efficacy of CB_2_ agonist is dependent upon CXCR4 signaling.

## Methods

### Subjects

One hundred and thirty-seven adult male Sprague–Dawley rats (Harlan, Indianapolis, IN, USA), weighing 305 to 400 g, were used in these experiments. All procedures were approved by Bloomington Institutional Animal Care and Use Committee (BIACUC) of Indiana University Bloomington and followed the guidelines for the treatment of animals of the International Association for the Study of Pain [68]. All animals were single housed in a temperature-controlled facility, with food and water *ad libitum*. Animals were maintained on a regular 12 h light/ 12 h dark cycle (lights on at 7 am).

### Drugs and chemicals

Cisplatin was purchased from Tocris Bioscience (Ellisville, MO, USA) and was dissolved in saline (0.9% sodium chloride). Paclitaxel was obtained from Tecoland Corporation (Edison, NJ, USA) and was dissolved in cremophor vehicle (1:1:4 ratio of cremophor^®^ EL/ ethanol/ saline). AM1710, AM630 and AM251 were synthesized by the Makriyannis laboratory. AMD3100 octahydrochloride hydrate was purchased from Sigma-Aldrich (St. Louis, MO, USA). AM1710, AM630, AM251 and AMD3100 were dissolved in 100% dimethyl sulfoxide (DMSO). DMSO, cremophor^®^ EL and acetone were purchased from Sigma-Aldrich (St. Louis, MO, USA). Saline was purchased from Aqualite System (Lake Forest, IL, USA).

### General experimental protocol

All experiments were conducted double-blinded with animals randomly assigned into groups. Cisplatin and paclitaxel were used to produce chemotherapy-induced neuropathy. Cisplatin (3 mg/kg i.p.) or saline vehicle was injected four times once weekly [69] in a volume of 10 ml/kg. Cisplatin/saline-treated animals were assessed for mechanical paw withdrawal thresholds and cold withdrawal frequencies every four days. Paclitaxel (2 mg/kg i.p.) or cremophor EL: ethanol: saline (1: 1: 4) vehicle was administered to rats four times every two days [70] in a volume of 1 ml/kg. Animals with paclitaxel/cremophor treatment were assessed for paw withdrawal thresholds to mechanical stimulation every two days and paw withdrawal frequencies to cold stimulation every four days. On the days animals received cisplatin/saline or paclitaxel/cremophor treatments, behavioral testing was performed prior to pharmacological manipulations.

Effects of pharmacological manipulations on mechanical and cold allodynia were assessed on day 28 in animals receiving cisplatin/saline treatments or day 20 in paclitaxel/cremophor-treated animals. On the test days, animals received either vehicle (DMSO), AM1710 either alone or in combination with the CB_2_ antagonist AM630 or the CB_1_ antagonist AM251, or the CXCR4 antagonist AMD3100. Withdrawal thresholds to mechanical stimulation and withdrawal frequencies to cold stimulation were measured before drug administration (−60 min) and at 30, 90, 150 min post drug administration in cisplatin/saline-treated animals, or at 30 min and 3 h post drug in paclitaxel/cremophor-treated animals. A subset of cisplatin- and paclitaxel-treated animals was additionally tested at 6 h and 24 h post drug administration.

In Experiments 1 and 2, antinociceptive effects of AM1710 in chemotherapy-induced neuropathy evoked by cisplatin or paclitaxel treatments were studied. Effects of AM1710 (0.1, 1 or 5 mg/kg i.p.) [19] or vehicle were assessed in animals receiving cisplatin or paclitaxel treatment. The high dose of AM1710 was also administered to animals that received saline or cremophor-vehicle in lieu of cisplatin or paclitaxel, respectively. To further evaluate the duration of action of the compound, a subset of paclitaxel-treated animals receiving AM1710 (5 mg/kg i.p.) or DMSO vehicle were tested from 30 min to 24 h post injection. Pharmacological specificity of anti-allodynic effects of AM1710 was assessed in both models by co-administering AM1710 (5 mg/kg i.p.) with the CB_2_ antagonist AM630 (3 mg/kg i.p.) [71] or CB_1_ antagonist AM251 (3 mg/kg i.p.) [72]. Separate groups received AM630 (3 mg/kg i.p.) or AM251 (3 mg/kg i.p.) alone. In Experiments 3 and 4, the CXCR4 antagonist AMD3100 (10 mg/kg i.p.) [73] was administered to animals to examine the impact of blockade of CXCR4 signaling on established neuropathy produced by cisplatin or paclitaxel treatment. AMD3100 (10 mg/kg i.p.) was administered to paclitaxel-treated animals either in absence or presence of AM1710 (5 mg/kg i.p.) to evaluate whether blockade of CXCR4 signaling would enhance CB_2_ agonist efficacy.

### Assessment of paw withdrawal thresholds to mechanical stimulation

Paw withdrawal thresholds to mechanical stimulation were measured using an electronic von Frey anesthesiometer (IITC model Alemo 2390–5, Woodland Hills, CA) as described previously [14]. Animals were placed on an elevated metal mesh table and habituated under inverted transparent plastic cages individually for at least 15 min prior to testing. After habituation to the testing environment (i.e. when animals ceased exploratory behaviors), a force was applied to the midplantar region of the hind paw by a rigid tip connected to the anethesiometer. Mechanical stimulation was terminated when the animal withdrew its paw and the value of the force was recorded in units of grams. Mechanical paw withdrawal thresholds were measured in duplicate for each paw and were reported as the mean of duplicate determinations averaged across paws.

### Assessment of paw withdrawal frequencies to cold stimulation

Paw withdrawal frequencies to cold stimulation were measured in the same animals assessed for mechanical hypersensitivity using the acetone method [74]. Rats were placed underneath inverted plastic cages on an elevated metal mesh table, the same environment used in the mechanical tests. After habituation, an acetone bubble that formed at the end of a blunt one C.C. syringe was gently presented to the plantar surface of the hind paw with care that application of acetone did not inadvertently result in mechanical stimulation of the paw with the syringe hub. Animals were observed for 20 seconds after acetone application. Acetone was applied to each paw of the animal 5 times alternately with a 3 min interval between applications. Paw withdrawal on a given trial was deemed present if animals showed one or more forms of unilateral nocifensive behavior. Trials on which an animal did not show unilateral behavior during the observation time were counted as zero. Unilateral behaviors that qualified as nocifensive behavior included withdrawing, raising, licking, shaking or repetitive stepping on the stimulated paw. Paw withdrawal frequencies were recorded as the percentage of the total number of occurrences of paw withdrawal out of 10 trials per animal.

### Statistical analyses

Paw withdrawal thresholds (mechanical) and frequencies (cold) were calculated for each paw and averaged. Analysis of variance (ANOVA) for repeated measures was used to determine the time course of paclitaxel and cisplatin-induced neuropathy as well as drug effects. One-way ANOVA was used to identify the source of significant interactions at each time point, followed by Dunnett *post hoc* tests (for comparisons to vehicle), Bonferroni *post hoc* tests (for comparisons between groups). A priori comparisons were also made using planned comparison and paired t-tests, as appropriate. All statistical analyses were performed using IBM-SPSS Statistics version 19.0 (SPSS inc., an IBM company, Chicago, IL, USA). *P* < 0.05 was considered statistically significant.

## Abbreviations

ANOVA: Analysis of variance; CB_1_: Cannabinoid receptor 1; CB_2_: Cannabinoid receptor 2; CCI: Chronic constriction injury; CNS: Central nervous system; CXCR4: Alpha-chemokine receptor; DMSO: Dimethylsulfoxide; DRG: Dorsal root ganglion; GLAST: Glutamate aspartate transporter; GLT-1: Glutamate transporter 1; HIV: Human immunodeficiency virus; i.p: Intraperitoneal; NMDA: N-methyl-D-aspartate; SDF-1/CXCL12: Chemokine stromal derived factor-1; s.c: Subcutaneous; TRPA: Transient receptor potential ankyrin; TRPV: Transient receptor potential vanilloid.

## Competing interests

The authors declared that they have no competing interest.

## Authors’ contributions

LD and JG contributed equally to this work. LD and JG conducted all behavioral tests, data analyses, and wrote the manuscript. KV synthesized AM630 and AM251 and GAT synthesized AM1710. AM630, AM251 and AM1710 were developed by AM. FAW contributed to the experimental design, provided AMD3100 used in an early study and revised the manuscript. AGH designed the studies, oversaw the project and wrote the manuscript. All authors read and approved the final manuscript.
